# The correlation of 25-hydroxyvitamin D with BMI and lipid profile: A retrospective cohort study of Syrian patients

**DOI:** 10.1016/j.amsu.2022.103457

**Published:** 2022-03-10

**Authors:** Sally Almatni, Mohammad Alsultan, Mohamed Taher Anan, Zaynab Alourfi

**Affiliations:** a Department of Endocrinology, Damascus University- Faculty of Medicine, Damascus, Syria; b Department of Nephrology, Damascus University- Faculty of Medicine, Damascus, Syria; c Department of Statics, Aleppo University- Faculty of Sciences, Aleppo, Syria

**Keywords:** BMI, 25OHD, LDL, TG, Cholesterol, Syria

## Abstract

**Background:**

This study aims to identify the prevalence of 25OHD deficiency in Syrian patients and investigate the relationship with obesity and lipid profile.

**Methods:**

A retrospective cohort study consisted of 201 patients of age >10 years, who referred to Al Assad and Al Mouwasat University Hospitals, Damascus, Syria from Oct/2020 to Oct/2021. The data was analyzed by using linear regressions and produced a matrix of correlations with significant equations between study variables.

**Results:**

Firstly, participants were divided depending on 25OHD levels, where 92.5% of patients had 25OHD <30 ng/mL. Inverse correlation between 25OHD and BMI (P ≤ 0.001) was observed, where severe 25OHD deficiency group had higher BMI (27.40 ± 7.22 kg/m^2^) and higher levels of Chol (211 ± 67.12 mg/dl) than in sufficiency group.Secondly, participants were divided depending on BMI. Higher BMI associated with lower levels of 25OHD. Moreover, we derived that every increase in 25OHD by 1 ng/mL results in decrease of BMI by 0.26 kg/m^2^ (P ≤ 0.001) and results in decrease of Chol by 1.54 mg/dl (P ≤ 0.004).

**Conclusion:**

A high prevalence of 25OHD deficiency was observed in this sample of Syrian patients. There is an inverse correlation between 25OHD and BMI regardless of age and gender. Moreover, the equation, that derived between 25OHD and BMI, represents a beneficial and an inexpensive tool in clinical practice to minimize testing of 25OHD by predicting its deficiency based on BMI and supports the impact of 25OHD supplementation for reduce BMI.

## Highlights

• 92.5% of **studied** patients had 25OHD levels <30 ng/mL and 72.1% of patients had 25OHD deficiency (<20 ng/mL).
• There is an inverse correlation between 25OHD and BMI regardless of age and gender.
• Severe 25OHD deficiency group (25OHD <10 ng/mL) had higher BMI and higher levels of Chol.
• Every increase in 25OHD by 1 ng/mL results in decrease of BMI by 0.26 kg/m^2^.
• Every increase in 25OHD by 1 mg/dl results in decrease of Chol by 1.54 mg/dl.

## 1 Introduction

The prevalence of overweight and obesity is increasing worldwide and this has raised serious public health concerns. Epidemiologic studies have identified high body mass index (BMI) as a risk factor for a wide range of chronic diseases, including cardiovascular disease [1,2], musculoskeletal disorders [3,4], diabetes mellitus, chronic kidney disease [1,5], infertility [6], asthma [7,8], sleep apnea, and many cancers such as esophageal, colon, endometrial and gall bladder [9].

Vitamin D has been the focus of interest in the past decade because, beyond of its known effects on skeletal system, data from epidemiological and observational studies have shown associations between low concentration of serum 25-hydroxyvitamin D (25OHD) and increased risk of many non-skeletal disorders [10]. A systematic review observed an association of low serum of 25OHD with cardiovascular diseases, serum lipid concentrations, glucose metabolism disorders, neurodegenerative diseases, infectious and inflammatory diseases, mood disorders, declining cognitive function and all-cause mortality [11].

Several theories were discussed to explain the relationship of 25OHD deficiency with BMI and lipid profile. People with high BMI usually have high-fat content, which represents a reservoir for lipid-soluble vitamin D [12]. Such individuals may need increasing vitamin D intake to maintain serum 25OHD levels comparable with individuals with normal BMI [13]. Another important function of vitamin D is the regulation of adiponectin production. This enzyme enhanced fatty acid oxidization and its levels have an inverse correlation with obesity and a positive correlation with vitamin D levels [14,15]. Probably, the most substantial mechanism is the volumetric dilution of 25OHD into greater tissue volume in obese and might need larger doses of 25OHD than normal weight to achieve the same serum levels [16,17].

With the high prevalence of vitamin D deficiency, which is estimated between 18% and 84% in various regions of the world [[18], [19], [20], [21]], several studies have been conducted to investigate the association of serum 25OHD with BMI and lipid profile [[22], [23], [24], [25]].

Since, a high proportion of 25OHD deficiency was seen in our daily practice, especially in obese; and limited studies were published from Syria, we proceed with this study to identify the prevalence of 25OHD deficiency in Syrian patients and investigate the relationship with obesity. The secondary goal is to study the influence of 25OHD deficiency on lipid profile, which represents by low-density lipoproteins (LDL), triglycerides (TG), and cholesterol (Chol).

## 2 Materials and methods

This retrospective cohort study consisted of 201 patients who referred as outpatient or admitted respectively to endocrinology clinic or department of Al Assad and Al Mouwasat University Hospitals, Damascus, Syria. Patients were collected from Oct/2020 to Oct/2021 and the study protocol was approved by the Research Ethics Committee of Damascus University and in accordance with the Declaration of Helsinki and in line with the STROCSS criteria [26].

Inclusion criteria was patients >10 years-old. Exclusion criteria was as follow: illness or disability limits precisely the measure of height and weight, received corticosteroids in the past three months, renal and hepatic failure, pregnant and breastfeeding females, gastrointestinal disease affects the absorption, and coincidence comorbidities influence especially on weight such as hypothyroidism, heart failure with edema, Cushing syndrome and tumors.

Data were collected by the same doctor along with all study period and included age, gender, BMI, 25(OH) vitamin D (25OHD), fasting plasma glucose (FPG) -after overnight fasting (at least 8–10 h)-, calcium (Ca), phosphorus (P), albumin (Alb) and lipid profile -after overnight fasting (at least ∼12 h)- which includes low-density lipoproteins (LDL), triglycerides (TG) and cholesterol (Chol). Height and weight were measured by endocrinologists residents and BMI was calculated by dividing weight (in kilograms) by height (in meters squared). BMI was classified as “underweight” (BMI <18.5 kg/m^2^), “normal weight” (BMI >18.5, and ≤25 kg/m^2^), “overweight” (BMI >25, and ≤30 kg/m^2^), “obesity class 1” (BMI >30, and ≤35 kg/m^2^), “obesity class 2” (BMI >35, and ≤40 kg/m^2^), and “obesity class 3” (BMI >40 kg/m^2^). BMI was classified for ages from 10 to 20 years as previous guidelines [27].

Total 25OHD levels were measured using automated electrochemiluminescence immunoassay (Elecsys 2010 analyzers, Roche Diagnostic Gmbh, Mannheim, Germany). Based on previous studies, Endocrine Society's recommendations and values classifications of our laboratory, 25OHD levels were classified as “severe deficiency” (25OHD <10 ng/mL), “deficiency” (25OHD ≥10, and <20 ng/mL), “insufficiency” (25OHD ≥20, and <30 ng/mL), and “sufficiency” (25OHD ≥30 ng/mL) level [28].

## 3 Statistical analysis

Statistical analysis was performed using program of R 4.02. We used nonparametric statistics such as Chi square test and parametric statistics such as one-tailed and two-tailed analysis of variance (ANOVA) and a measure of linear correlation using the Pearson correlation coefficient. Descriptive analysis was performed using mean and standard deviation (mean ± SD). Also, we used simple and multiple linear regression to build the models between study variables. *P*-value < 0.05 was considered statistically significant.

## 4 Results

Patients characteristics are presented in Table 1. Of 201 patients, 76 (37.81%) were male and 125 (62.18%) were female with mean age (37.58 ± 17.27), mean serum 25OHD was (14.59 ± 8.87 ng/mL) and mean BMI was (24.75 ± 6.97 kg/m^2^).

**Table 1:** Patients characteristics.

Variable	Number	Minimum	Maximum	Mean	± SD
Age	199	11	82	37.58	17.27
25OHD (ng/mL)	201	2.60	51	14.59	8.87
BMI (kg/m^2)^	201	14.20	52.80	24.75	6.97
Chol (mg/dL)	144	81	374	186	61.81
LDL (mg/dL)	128	23	294	89.87	43.18
TG (mg/dL)	119	27	291	142	61.76
Ca (mg/dL)	116	5.10	10.50	8.95	0.76
P (mg/dL)	81	1.60	6	3.81	0.97
Alb (mg/dL)	86	2.50	5.40	4.18	0.50
Glu (mg/dL)	42	70	289	125	56.22

SD; standard deviation, 25OHD; 25(OH) vitamin D, BMI; body mass index, Chol; cholesterol, LDL; low-density lipoproteins, TG; triglycerides, P; phosphorus, Ca; calcium, Alb; albumin, Glu; glucose.

Firstly, study participants were divided into four groups depending on 25OHD levels and were analyzed with rest variables (Table 2). Age and gender have no statistically significant between groups (P = 0.24 and 0.43; respectively). Mean levels of 25OHD in all age groups located under deficient levels (<20 ng/mL).

**Table 2:** Correlation between 25OHD ranges and other variables.

Gender	Age	25OHD (mean ± SD)				P. value
Male N = 75	10–25	N = 25 (14.85 ± 9.02)				Age = 0.24 Gender = 0.43
	25–50	N = 32 (15.48 ± 10.56)				
	˃ 50	N = 18 (14.83 ± 8.63)				
Female N = 124	10–25	N = 35 (11.58 ± 6.68)				
	25–50	N = 56 (15.11 ± 8.58)				
	˃ 50	N = 33 (15.40 ± 9.77)				
**Variable**	**Total Number**	**25OHD**				
		˂ 10	10–20	20–30	˃ 30	
		N Mean ± SD	N Mean ± SD	N Mean ± SD	N Mean ± SD	
**BMI**	201	N = 85 27.40 ± 7.22	N = 60 24.33 ± 6.39	N = 41 20.98 ± 5.24	N = 15 21.71 ± 5.98	0.000
**Chol**	144	N = 69 211 ± 67.12	N = 33 161 ± 37.22	N = 29 155 ± 38.15	N = 13 186 ± 73.28	0.0
**LDL**	128	N = 62 96.43 ± 37.38	N = 28 89.53 ± 37.92	N = 28 73.32 ± 39.17	N = 10 96.50 ± 82.01	0.122
**TG**	118	N = 63 30.92 ± 25.32	N = 19 43.10 ± 33.26	N = 25 55.56 ± 33.86	N = 11 41.45 ± 32.12	0.006
**Glu**	42	N = 16 121 ± 44.77	N = 15 128 ± 64.28	N = 6 125 ± 76.11	N = 5 131 ± 54.92	0.80
**Ca**	116	N = 44 8.40 ± 0.78	N = 36 8.50 ± 0.94	N = 27 8.77 ± 0.75	N = 9 8.66 ± 0.50	0.30
**P**	81	N = 33 3.27 ± 1.15	N = 25 3.36 ± 0.90	N = 18 3.88 ± 0.96	N = 5 3.20 ± 0.44	0.191
**Alb**	86	N = 31 4.08 ± 0.53	N = 28 4.18 ± 0.52	N = 21 4.25 ± 0.40	N = 6 4.41 ± 0.59	0.41

Only 15 patients (7.5%) had sufficient levels of 25OHD (≥30 ng/mL) and 25OHD <30 ng/mL was reported in 186 patients (92.5%). Subsequently, 72.1% of patients (n = 145) had 25OHD deficiency (<20 ng/mL) and 20.4% of patients (n = 41) had 25OHD insufficiency (≥20 and < 30 ng/mL).

BMI, Chol and TG have statistically significant differences between groups (P; ≤ 0.001, ≤0.01 and ≤ 0.006; respectively). On the other hand, there is no statistically significant differences in LDL.

There is statistically significant inverse correlation observed between 25OHD and BMI (P ≤ 0.001). Of 201 patients, 85 patients in severe deficiency group (25OHD <10 ng/mL) have overweight BMI (27.40 ± 7.22 kg/m^2^), which higher than BMI (21.71 ± 5.98 kg/m^2^) in sufficiency group (25OHD ≥30 ng/mL). Also, patients in severe deficiency group had higher levels of Chol (211 ± 67.12 mg/dl) than Chol levels (186 ± 73.28 mg/dl) in sufficiency group.

Secondly, study participants were divided into five groups depending on BMI classifications and were analyzed with rest variables (Table 3). Gender had no statically significance (P = 0.160) but mean BMI in female (25.10 ± 7.22 kg/m^2^) was higher than male (23.67 ± 6.57 kg/m^2^). Age shows statistically significant (P ≤ 0.006) only in male group, but higher age associates with higher levels of BMI, in both genders. There is statistically significant inverse correlation was observed between 25OHD and BMI (P ≤ 0.001), where higher BMI associates with lower levels of 25OHD, and the lowest levels of 25OHD (9.96 ± 6.10 ng/mL) observe in obesity class 1 group. Also mean levels of 25OHD in all BMI groups located under deficient levels (<20 ng/mL).

**Table 3:** Correlation between BMI ranges and other variables.

Variable	Total Number							P. value
Gender	N = 201	Male, N = 76 Mean BMI ±SD = 23.67 ± 6.57			Female, N = 125 Mean BMI ±SD = 25.10 ± 7.22			0.160
		BMI						
		˂ 18.5	18.5–25	25–30	30–35	35–40	˃ 40	
		Mean ± SD	Mean ± SD	Mean ± SD	Mean ± SD	Mean ± SD	Mean ± SD	
Age	M = 75	N = 18 23.16 ± 13.14	N = 25 39.84 ± 19.41	N = 21 39.19 ± 16.49	N = 7 48.42 ± 13.64	N = 3 43.66 ± 11.93	N = 1 47	0.006
	F = 124	N = 22 31.27 ± 18.24	N = 45 35.93 ± 16.68	N = 25 38.72 ± 16.21	N = 21 46.38 ± 13.36	N = 6 42.33 ± 21.36	N = 5 45.40 ± 16.33	0.057
25OHD	201	N = 42 18.32 ± 8.94	N = 69 17.65 ± 9.52	N = 47 10.42 ± 5.52	N = 28 9.96 ± 6.10	N = 9 11.86 ± 11.00	N = 6 11.73 ± 5.77	0.000
Chol	144	N = 31 164 ± 54.84	N = 42 185 ± 54.36	N = 36 186 ± 64.37	N = 22 234 ± 68.00	N = 8 140 ± 28.57	N = 5 188 ± 36.44	0.00
LDL	128	N = 27 80.29 ± 37.16	N = 37 76.59 ± 39.28	N = 30 87.60 ± 36.56	N = 21 125 ± 55.15	N = 8 87.87 ± 27.34	N = 5 108 ± 29.49	0.001
TG	118	N = 24 52.04 ± 32.19	N = 38 33.95 ± 30.24	N = 32 38.34 ± 30.49	N = 17 30.82 ± 25.64	N = 7 46.86 ± 28.80	N = 1 17.0	0.172
Glu	42	N = 10 104 ± 33.65	N = 20 118 ± 41.51	N = 10 133 ± 67.15	N = 3 203 ± 110.01	___	___	0.04
Ca	116	N = 26 8.57 ± 0.85	N = 30 8.76 ± 1.07	N = 32 8.46 ± 0.50	N = 14 8.35 ± 0.84	N = 9 8.33 ± 0.70	N = 5 8.40 ± 0.54	0.553
P	81	N = 19 3.73 ± 0.99	N = 18 3.50 ± 0.85	N = 22 3.18 ± 0.79	N = 12 3.41 ± 0.99	N = 7 3.42 ± 2.07	N = 3 3.00 ± 0	0.616
Alb	86	N = 17 4.34 ± 0.44	N = 21 4.36 ± 0.48	N = 24 4.01 ± 0.57	N = 14 4.03 ± 0.51	N = 6 4.15 ± 0.25	N = 4 4.10 ± 0.27	0.135

Moreover, there are statistically significant differences in LDL, Chol, and Glu between groups (P ≤ 0.001, ≤0.01; and ≤0.04; respectively). Of 144 patients, Chol levels were lowest (164 ± 54.84 mg/dl) in underweight group, and highest Chol levels (234 ± 68.00 mg/dl) were in obesity class 1 group. Also, of 128 patients, LDL levels were lower (80.29 ± 37.16 mg/dl) in underweight group than LDL levels (125 ± 55.15 mg/dl) in obesity class 1 group.

The matrix of correlations between study variables using two tailed analysis test shows in (Table 4) and produces a statically significant equations (Table 5). This matrix shows a direct correlation of positive values and inverse correlation of negative values. Statically significant inverse correlation was observed between BMI and 25OHD (P ≤ 0.001), also a direct correlation of BMI with LDL and Glu was observed of statically significant (P ≤ 0.001; ≤0.001; respectively). 25OHD has a statically significant inverse correlations with Chol (P ≤ 0.004) and a direct correlation with TG (P ≤ 0.016).

**Table 4:** Matrix of correlations between study variables.

	Age	BMI	25OHD	Chol	LDL	TG	Ca	P	ALb	GLU
Age p-value	1	.282∗∗	.062	.027	.050	-.020	.012	-.336∗∗	-.334∗∗	-.168
		.000	.384	.753	.579	.830	.899	.002	.002	.288
BMI p-value		1	-.337∗∗	.147	.288∗∗	-.120	-.120	-.190	-.228∗	.515∗∗
			.000	.078	.001	.192	.200	.090	.035	.000
25OHD p-value			1	-.237∗∗	-.128	.220∗	.154	.135	.229∗	-.407∗∗
				.004	.151	.016	.099	.231	.034	.008
Chol p-value				1	.462∗∗	-.433∗∗	.176	.020	.181	.753∗∗
					.000	.000	.095	.869	.133	.000
LDL p-value					1	-.219∗	.150	.097	.249∗	.610∗∗
						.023	.188	.457	.048	.001
TG p-value						1	-.164	.192	-.186	-.265
							.180	.169	.179	.156
Ca p-value							1	-.154	.448∗∗	.083
								.170	.000	.779
P p-value								1	.115	.192
									.359	.621
ALb p-value									1	.289
										.451
GLU p-value										1
										42

Positive values mean a direct correlation between variables.

Negative values mean an inverse correlation between variables.

∗ Correlation is significant at the 0.05 level (2-tailed).

∗∗ Correlation is significant at the 0.01 level (2-tailed).

**Table 5:** Models derived from the matrix between study variables.

Equations between variables∗	*P*- value
BMI = 28.45 - 0.26 (25OHD)	0.000
BMI = 20.76 + 0.05 LDL	0.001
BMI = 41.015–3.60 Alb	0.03
BMI = 17.15 + 0.40 Glu	0.000
Chol = 208.75 - 1.54 (25OHD)	0.004
TG = 29.12 + 0.70 (25OHD)	0.016
Alb = 3.99 + 0.013 (25OHD)	0.034
Glu = l65.23 - 2.34 (25OHD)	0.008

∗ Statically significant equations between variables.

From matrix equations (Table 5), we can derive that every increase in 25OHD by 1 ng/mL results in decrease of BMI by 0.26 kg/m^2^ (P ≤ 0.001). Also, we can derive that every increase in 25OHD by 1 mg/dl results in decrease of Chol by 1.54 mg/dl (P ≤ 0.004). A direct correlation between 25OHD and TG was observed, that every increase in 25OHD by 1 mg/dl results in increase of TG by 0.70 mg/dl (P ≤ 0.016).

Based on all previous tests, there is a strong and statically significant correlation between 25OHD deficiency and BMI.

## 5 Discussion

This retrospective cohort study of Syrian patients accounts a high proportion of 25OHD deficiency (72.1%) regardless of age or gender. This study concludes a strong inverse correlation between 25OHD and BMI. Also, the relationship of 25OHD deficiency with lipid profile shows a statically significant correlation with Chol and TG but no relation with LDL.

The relationship between 25OHD deficiency and obesity has widely been studied. A meta-analysis confirmed that obesity associated with vitamin D deficiency regardless of different age groups including studies among child participants, and reported a 35% higher prevalence of vitamin D deficiency in obese compared to the eutrophic. In addition to suggest the necessity to monitor 25OHD levels among obese individuals [24]. Another study finds a high prevalence (85%) of obese adults, in the US, have serum 25OHD levels less than 30 ng/mL [25].

Our findings correspond with previous studies in reporting the association between 25OHD and obesity regardless of age and gender. Moreover, the present study confirmed the inverse correlation between BMI and 25OHD by classifying the population based on 25OHD levels and then based on BMI levels, which showed lower levels of 25OHD in obese patients especially in obesity class 1 group (BMI between 30 and 35 kg/m^2^) (Table 3). A higher prevalence (92.5%) of 25OHD <30 ng/mL was reported in study participants, this might due to poor living and diet situations. Additionally, we derive a simple equation between these two disorders, that every increase in 25OHD by 1 ng/mL results in decrease of BMI by 0.26 kg/m^2^ (P ≤ 0.001) (Table 5). This might be a useful and an inexpensive tool in clinical practice to predict and subsequently monitor the 25OHD levels based on BMI.

Dyslipidemia has shown an association with vitamin D deficiency in many studies [29]. Rates of dyslipidemia in vitamin D deficiency, insufficiency, and sufficiency groups were 45.4%, 41.6%, 38.8%, respectively [29]. A meta-analysis of 21 studies mentioned that the majority of results indicated inversely relations of serum 25OHD with Chol, LDL, and TG and direct correlation with serum HDL in children and adolescents [23].

However, the current study finds no significant relation between 25OHD and LDL, an inverse correlation of 25OHD with Chol and a direct correlation with TG, which differing from previous studies. We conclude that every increase in 25OHD by 1 mg/dl results in decrease of Chol by 1.54 mg/dl and increase of TG by 0.70 mg/dl. Also, highest levels of Chol was observed in severe deficiency patients (25OHD <10 ng/mL), and in obesity class 1 group (BMI between 30 and 35 kg/m^2^) (Table 3).

Previous studies appeared a beneficial impact of vitamin D supplementation for obesity but the result with dyslipidemia were conflict, however, the follow-up period was short [30,31]. A meta-analysis of 11 RCTs, lasting between 1 and 12 months, revealed that vitamin D supplementation has a desirable effect on weight loss by reducing BMI in overweight and obese individuals [30]. Another meta-analysis of 41 studies in adults, lasting for a year in maximum, revealed that vitamin D supplementation has a beneficial effect on reducing serum total Chol, LDL, and TG levels but not HDL levels [31]. On the other hand, conflict results of a meta-analysis of 17 studies showed a higher serum 25OHD related to a more favorable lipid profile only in the pediatric group [23].

The statically significant equations of 25OHD with BMI and Chol, derived in this study, support the impact of 25OHD supplementation for reduce BMI and Chol. In different to previous studies, a direct correlation of 25OHD with TG was observed in our patients and this might due to different aged participants and different diets compared to other regions or drugs that affect TG levels.

However, the adequate dosage and duration of this supplementation are still unclear [30], and which levels of serum 25OHD should be achieved to serve as a biomarker of health outcomes is also undefined [13].

Limitations of this study were the small number of participants to account for the real prevalence of 25OHD deficiency in the Syrian population, HDL was not studied and the impact of several factors such as dietary intake and physical activity were not included. The cutoff age for admission in mentioned hospitals is limited to above 10 years old. Also, there were no available materials for testing lipid profile or 25OHD for several months in the past year because of limited resources in our hospitals. In addition to excluding several comorbidities that might influence weight. These reasons reduced the number of patients included in the past year.

## 6 Conclusion

A high prevalence of 25OHD deficiency was observed in this sample of Syrian patients. There is an inverse correlation between 25OHD and BMI regardless of age and gender, and the lowest levels of 25OHD shows in obesity class 1 group. Moreover, the equation, that derived between BMI and 25OHD, represents a beneficial and inexpensive tool in clinical practice to predict 25OHD deficiency based on BMI and supports the impact of 25OHD supplementation for reduce BMI. Also, the highest level of Chol was observed in severe 25OHD deficiency patients. These data suggest the necessity to measure serum 25OHD in obese patients, and subsequently researches are needed to identify the cut-off point for 25OHD levels and its impact in obesity and general health outcomes.

## Ethical approval

This study protocol was reviewed and approved by Ethics Committee of the Damascus University Research Centre and with the Helsinki declaration.

## Sources of funding

This research did not receive any specific Grant from funding agencies in the public, commercial, or not-for-profit sectors.

## Author contributions

Dr. Sally Almatni wrote the manuscript, literature search, and submitted the article.

Dr. Mohammad Alsultan wrote the manuscript and literature search.

Dr. Mohamed Taher Anan; data analysis, wrote and explain the study results.

Dr. Zaynab Alourfi; made study corrections and supervisor of the research.

## Trail registry number

1. Name of the registry: OSF Preregistration.

2. Unique Identifying number or registration ID: osf.io/98aju

3. Hyperlink to your specific registration (must be publicly accessible and will be checked): https://archive.org/details/osf-registrations-98aju-v1.

## Guarantor

Dr. Sally Almatni.

## Consent

Written informed consent was obtained from patients for publication of this article and any accompanying images.

## Declaration of competing interest

The author declares that they have no conflicts of interest regarding this study.

The author declares that it has not been published elsewhere and that it has not been submitted simultaneously for publication elsewhere.
